# Therapeutic Effect of the Mitochondria-Targeted Antioxidant SkQ1 on the Culture Model of Multiple Sclerosis

**DOI:** 10.1155/2019/2082561

**Published:** 2019-07-01

**Authors:** Elena K. Fetisova, Maria S. Muntyan, Konstantin G. Lyamzaev, Boris V. Chernyak

**Affiliations:** A.N. Belozersky Institute of Physico-Chemical Biology, Lomonosov Moscow State University, Moscow, Russia

## Abstract

Multiple sclerosis (MS) is a heterogeneous autoimmune disease of unknown etiology characterized by inflammation, demyelination, and axonal degeneration that affects both the white and gray matter of CNS. Recent large-scale epidemiological and genomic studies identified several genetic and environmental risk factors for the disease. Among them are environmental factors of infectious origin, possibly causing MS, which include Epstein-Barr virus infection, reactivation of some endogenous retrovirus groups, and infection by pathogenic bacteria (mycobacteria, *Chlamydia pneumoniae*, and *Helicobacter pylori*). However, the nature of the events leading to the activation of immune cells in MS is mostly unknown and there is no effective therapy against the disease. Amazingly, whatever the cause of the disease, signs of damage to the nerve tissue with MS lesions were the same as with infectious leprosy, while in the latter case nitrozooxidative stress was suggested as the main cause of the nerve damage. With this in mind and following the hypothesis that excessive production of mitochondrial reactive oxygen species critically contributes to MS pathogenesis, we studied the effect of mitochondria-targeted antioxidant SkQ1 in an *in vitro* MS model of the primary oligodendrocyte culture of the cerebellum, challenged with lipopolysaccharide (LPS). SkQ1 was found to accumulate in the mitochondria of oligodendrocytes and microglial cells, and it was also found to prevent LPS-induced inhibition of myelin production in oligodendrocytes. The results implicate that mitochondria-targeted antioxidants could be promising candidates as components of a combined therapy for MS and related neurological disorders.

## 1. Introduction

Typical multiple sclerosis (MS) is a widespread chronic inflammatory demyelinating disease of the central nervous system (CNS), a hallmark of which is considered the demyelination of cerebral white matter and a consequent progressive neuronal loss and neurological disability. The etiology of MS is multifactorial and usually associated with autoimmune processes, but it remains not clear as of now [1]. During the last decades, a significant body of data indicated a critical contribution of mitochondria and oxidative stress to both inflammatory and neurodegenerative aspects of MS pathogenesis [2]. The recent observation of the sharp appearance of the free mitochondrial DNA in the serum of patients with MS at the onset of the disease also supports this view [3]. In this context, it should be noted that myelin-producing glial cells called oligodendrocytes are particularly susceptible to oxidative stress and inflammatory mediators [4–6] and are one of the main targets of MS. All of these led us to suggest that mitochondria-targeted antioxidants (mtAO), detoxifying mitochondrial reactive oxygen species (mtROS), could be promising components of combined programs for MS therapy. Among mtAO, the most widely used is MitoQ 10-(6′-ubiquinonyl) decyltriphenylphosphonium bromide, which has been shown to inhibit oxidative stress in nervous tissue in various animal models of neurological diseases, including Alzheimer's [7] and Parkinson's [8] diseases, as well as amyotrophic lateral sclerosis [9]. Application of MitoQ to experimental autoimmune encephalomyelitis (EAE), a mouse model of MS, demonstrated an increase in myelin basic protein (MBP) production and attenuation of neurodegeneration [10]. Developed in 2008, mtAO compounds of the SkQ family (conjugates of plastoquinol with various penetrating cations [11, 12]) were found to be more effective than MitoQ in various in vitro models of diseases. The pronounced protective effects of SkQ1 (10-(6′-plastoquinonyl) decyltriphenylphosphonium) and its analogs were observed in the model of open focal trauma of the sensorimotor cortex [13] and in the model of brain ischemia/reperfusion injury [14]. SkQ1 significantly improved neurological deficits in the senescence-accelerated OXYS rats, which in various aspects resembled symptoms of Alzheimer's disease. The long-term treatment with SkQ1 slowed down the pathological accumulation of beta-amyloid and the hyperphosphorylation of tau protein (markers of Alzheimer's disease pathogenesis) in the cortex and hippocampus of OXYS rats [15, 16].

In MS therapy, the protection of oligodendrocytes from oxidative stress could be a key strategy that can ensure the maturation of immature oligodendrocytes and their remyelinization, as well as increase the chances of improving the neurological state of patients [17]. In the present study, we made the first step to test the effect of SkQ1 on oligodendrocytes *in vitro* and used for this purpose the primary oligodendrocyte culture that migrated from cerebellar explants, which was challenged with lipopolysaccharide (LPS). This culture also contains microglia [18], in which the effects of LPS are mediated in at least two ways: (i) at low LPS concentrations (1 ng/ml) by specific toll-like receptor TLR4, which is not expressed in oligodendrocytes [19, 20], and (ii) at LPS concentrations two or three orders of magnitude higher by a TLR4-independent pathway [21]. An important advantage of the used culture model is the preservation of conditions for the survival (and maturation) of oligodendrocyte progenitor cells, as well as mature myelinating oligodendrocytes. The challenge of the culture with LPS recapitulates various features of brain inflammation including oxidative stress, demyelination, and axonal damage [22, 23]. These features of the used primary oligodendrocyte culture of cerebellar explants make it suitable for the examination of various agents affecting the myelination/demyelination of oligodendrocytes. In the present study, we demonstrated that SkQ1 in as low as nanomolar concentrations significantly inhibited an LPS-induced decrease in myelin content in oligodendrocytes.

## 2. Materials and Methods

### 2.1. Reagents

LPS from *E. coli* (# 055:B5) was purchased from Sigma-Aldrich (USA). Mitochondria-targeted antioxidant SkQ1 (10-(6′-plastoquinonyl) decyltriphenylphosphonium bromide) was synthesized as described earlier [11] and kindly provided by the Institute of Mitoengineering, Lomonosov Moscow State University. For immunostaining, the following antibodies were used: rabbit polyclonal anti-beta III tubulin antibody (Abcam, ab76287), a neuronal marker; mouse anti-Oligo1 monoclonal antibody (# MAB5540; Millipore, USA) against oligodendrocyte transcription factor; antibody against myelin basic protein (MBP) (# M3821 Sigma-Aldrich); anti-iNOS antibody against inducible NO-synthase (Abcam, ab3523), and Alexa Fluor 488-conjugated anti-IgG (Invitrogen, USA).

### 2.2. Preparation of the Primary Oligodendrocyte Cell Cultures

The primary oligodendrocyte cell cultures were formed by the cells that migrated from the cerebellar explants which were prepared from the brains of newborn rats (1–2 days old) and cultivated according to the method of Viktorov and Kernarskaya [24]. Cerebellum fragments (1 × 1 mm) were plated upon poly-D-lysine-coated, 5 *μ*g/ml (Sigma-Aldrich) coverslips placed inside Petri dishes filled with nutrient medium where the cultivation was performed at 37°C in an air atmosphere containing 5% CO_2_. The nutrient medium was composed of DMEM containing glucose 4.5 g/l (Dulbecco's Modified Eagle's Medium) and F12 (Ham's Medium) at a ratio of 1 : 1 (*v*/*v*), supplemented with 24% fetal bovine sera, 9% embrional chicken extract, and 5 mM glutamine, where all reagents and solutions were from PanEco, Russia. Additionally, the nutrient medium was supplemented with 10 *μ*M acetylsalicylic acid (Sigma-Aldrich), 0.5 *μ*g/ml insulin (Sigma-Aldrich), streptomycin (100 U/ml), and penicillin (100 U/ml). Every 2-3 days, the medium was replaced for a fresh one.

### 2.3. Investigation of the Primary Oligodendrocyte Cell Cultures

Myelin synthesis by oligodendrocytes was analyzed by probing the cultures with rabbit antibody to MBP. Cultures were treated with 5 nM SkQ1 solution for 10 days and with 10 nM SkQ1 solution for the last 2 days of cultivation. LPS (5 *μ*g/ml) was added from the third day of cultivation for 10 days and removed for the last 2 days of cultivation.

### 2.4. Immunostaining

Before immunostaining, cells grown on glass coverslips were fixed with 1% formaldehyde in phosphate-buffered saline solution (PBS) for 1.5 hours at room temperature to ensure thorough fixation essential for preventing the fragments from being detached. Thereafter, these preparations were rinsed with PBS and initially stained with the following antibodies: anti-Oligo1 antibody for oligodendrocytes, anti-beta III tubulin antibody for neurons, antibody against MBP, and antibody against iNOS. Alexa-Fluor-488-conjugated secondary antibodies were used after processing with the primary antibodies. Immunofluorescence was observed and analyzed with an LSM 510 confocal microscope (Carl Zeiss, Germany) using optical filters “555 nm” (red) and “488 nm” (green).

### 2.5. Statistical Analysis

For statistical analyses, one-way ANOVA followed by Bonferroni's test was performed using GraphPad Prism version 6.01 for Windows (GraphPad Software, La Jolla California USA, http://www.graphpad.com). A *p* value of <0.05 was considered statistically significant.

## 3. Results and Discussion

**Figure 1:**
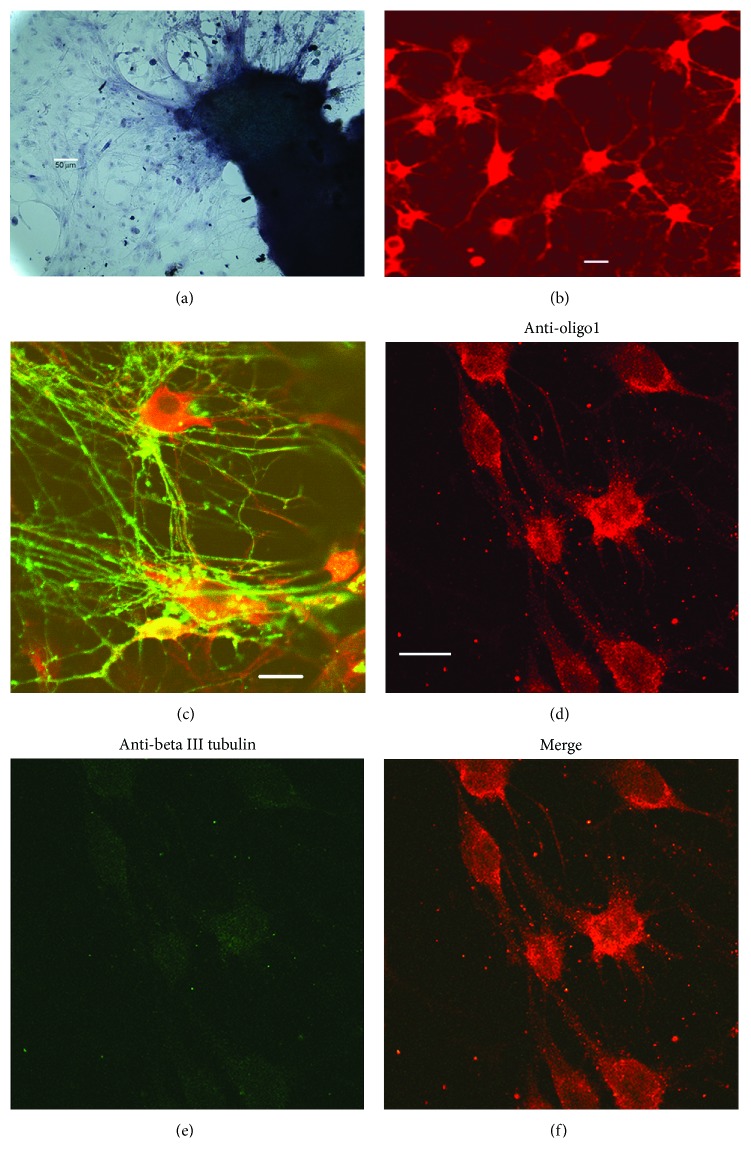
Determination of the cell types forming the migration zone in the primary oligodendrocyte culture of cerebellar explants upon 10 days of cultivation. (a) Cultured oligodendrocytes of a newborn rat: general view of the culture. The cells radially migrate from the explant of the cerebellum (dark-gray fragment on the right side) *in vitro*. Bar, 50 *μ*m; transmitted-light microscopy; objective—10x. (b) The field of view within the formed migration zone. The cells were probed with anti-Oligo1 antibody. (c) Myelin synthesis by oligodendrocytes; merged image; probing with both antibody types, anti-Oligo1 to mark oligodendrocytes (red) and antibodies to MBP to mark myelin (green). (d–f) The cells were probed with both types of antibodies, anti-Oligo1 antibody for oligodendrocytes (positive result, red) and anti-beta III tubulin antibody for neurons (negative result, green). (b–f) Bar, 10 *μ*m; confocal microscope, Carl Zeiss LSM 510.

**Figure 2:**
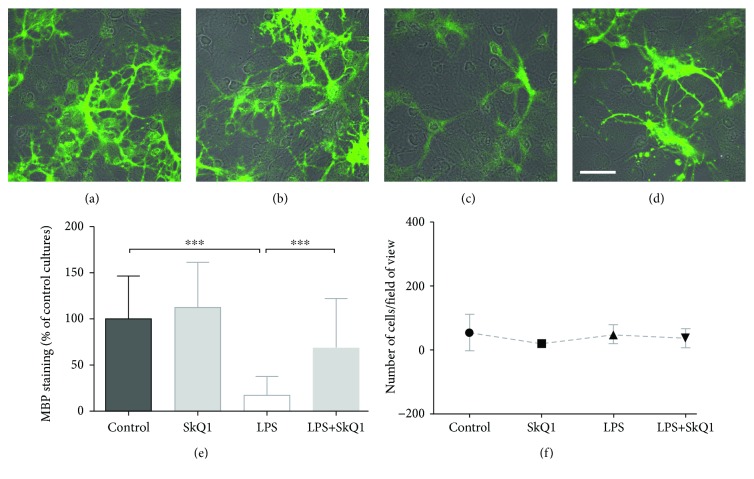
SkQ1 protects against the LPS-induced decrease in myelin content in oligodendrocytes. (a–d) Representative images of immunostaining for MBP: (a) control and treatment with the reagents (b) SkQ1, (c) LPS, and (d) LPS+SkQ1. (e) MBP staining was quantified, measuring 7-60 fields of view per treatment and expressed as the stained area percentage of untreated control cultures. (f) The number of cells in the field of view. Samples were prepared from the brain of eight newborn rats in seven independent experiments. Data are expressed as mean ± SD (^∗∗∗^ *p* < 0.0003; one-way ANOVA, Bonferroni's post hoc test). Scale bar, 20 *μ*m.

In the present study, we used the explant culture of newborn rat cerebellum (1) for the preparation the primary culture of oligodendrocytes (2), both cultures, (1) and (2), being formed on the same glass coverslip (Figure 1(a)). The primary culture of oligodendrocytes in this case is formed in the process of emigration of cells from the explant for 10-14 days, which leads to the formation of a cell monolayer on the same coverslip as can be seen by staining the cultures with the specific antibody, the marker of oligodendrocytes, Oligo1 (Figures 1(b)–1(d) and 1(f)). Oligodendroglia is the first to emigrate from the explant due to the mobility of oligodendrocytes and the ability of cells to change their shape and size, which was well documented by several authors [18, 25, 26]. In our study, we confirm these observations of the mentioned authors and demonstrate that the first emigrants formed the cell layer presented practically only by oligodendrocytes (Figures 1(b)–1(f)). Neither neuron cells nor other types of cells were detected in these monolayers. The positively Oligo1-stained cells of irregular shape and relatively large sizes were forming the cell-to-cell chains characteristic of oligodendrocytes, which resemble “being engaged in a tug-of-war” [26]. The barely perceptible “green” signal (Figure 1(e)) on staining with the anti-beta III tubulin antibody (marker of neurons) fully coincides with the bright “red” signal of anti-Oligo1 identifying only oligodendrocytes present in the field. Thus, the poor “green” signal is the background staining and the absence of bright “green” demonstrates the lack of neurons in the monolayer field of the migration zone in accordance with the observations made earlier [26, 27]. Moreover, the absence of the “green” background staining shows as well the lack of any other cell types in this migration zone which apparently is inhabited mainly by oligodendrocytes. However, the primary explant culture of oligodendrocytes is formed on the same surface as the cerebellar explant itself. Note that although cultures (1) and (2) are spatially separated as they are located on the different parts of the coverslip, the layer of the emigrated cells and the parent explant are covered by the common thin layer of the liquid nutrient medium. Consequently, all the cell types that inhabit the coverslip can exchange metabolites among themselves. This means that oxidative stress factors are common to all cells of each coverslip, each of which, accordingly, in general, contains a total set of neuroglial cells represented in the cerebellum: neurons, astrocytes, oligodendrocytes, and microglia. In such tissue type, on average, the number of neuroglia is 8-10 times more than that of neuron cells [28, 29]. As shown in Figure 1(c), upon 10 days of cultivation, a regular myelin synthesis (green) was observed in the processes of cultured oligodendrocytes (red). Within the 10–14-day period, when oligodendrocytes and their processes were visualized as a dense green network due to abundantly synthesized myelin (anti-MBP staining; see Figure 2(a) and Supplementary Figures 1(a) and 1(c)), investigation of oligodendrocyte demyelination/remyelination was carried out.

**Figure 3:**
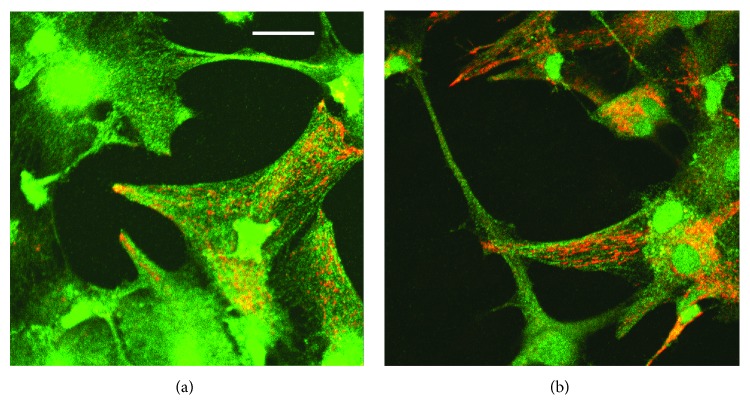
Mitochondria-targeted antioxidant SkQR1, a red-colored fluorescent SkQ derivative, selectively accumulates in mitochondria (orange) of glial cells: (a) oligodendrocytes and (b) microglial cells. The primary oligodendrocyte culture of cerebellar explants was preincubated with both (a, b) 50 nM SkQR1 for 2 h and (a) anti-Oligo1 antibody for staining oligodendrocytes (green) or (b) anti-iNOS antibody (green). Images are the merge of views with “red” and “green” optical filters. Scale bar, 5 *μ*m. Confocal microscope, Carl Zeiss LSM 510.

Prior to investigating demyelination/remyelination, we studied the antioxidant penetration in the cultured cells. To analyze localization of SkQ1 in oligodendrocytes, we used the fluorescent SkQ1 analog, SkQR1, in which the cationic constituent, triphenylphoshonium, was replaced by cation rhodamine-19. According to their antioxidant protective properties, both analogs are among the most effective in the SkQ family and are of the same level of penetrating ability for model lipid membranes [11, 30] and mitochondria of both various cell types *in vitro* [11] and some tissues *in vivo* [31]. As to their antioxidant property, the protective antioxidant effect of SkQR1 compared to SkQ1 was slightly higher in some of the models *in vitro* [11] and *in vivo* [31, 32]. Mitochondrial localization of SkQR1 was earlier demonstrated in various cell types, such as HeLa cells [11], endothelial cells of the EaHy926 cell line [33], and human neutrophils [34], as well as human skin fibroblasts [35] and myofibroblasts [36]. Like in the other cell types, in oligodendrocytes migrating from cerebellar explants, SkQR1 selectively accumulated in mitochondria that formed the well-recognized mitochondrial network, shown both during this study (Figure 3(a)) and in previous examinations (Figure 1 in ref. [2]).

Due to natural causes, some cells inside the explant died during the cultivation and microglial cells were visible in the space adjacent to the cerebellar explant.  Figure 3(b) shows a dense mitochondrial network (orange) that pierces microglial cells visible in the explant cerebellar cultures stained with anti-iNOS antibody (green) and mitochondria-targeted antioxidant SkQR1.

To initiate neuroinflammation in the explant culture, we used LPS in high concentration (5 *μ*g/ml) in the presence of serum which was a component of the cultivation medium. A strong LPS-induced decrease in the myelin content in oligodendrocytes was observed (Figure 2(c) and Supplementary Figures 1(b) and 1(d)). Under these conditions, the effect of LPS on microglia is mediated by both TLR4-dependent and TLR4-independent pathways as described earlier [21]. The direct effects of LPS on oligodendrocytes also cannot be excluded [37, 38]. The pretreatment of the culture with SkQ1 in nanomolar concentrations significantly protected oligodendrocytes (Figure 2(d)). The decrease and disappearance of staining for myelin were not the result of cell death in the culture, because in all experiments the number of cells in the samples remained constant (Figure 2(f)).

This result indicates an important contribution of glial mitochondria to the total oxidative burst caused by LPS, if we take into account the experimental data on the mechanism of SkQ1 action [39]. SkQ1 is a lipophilic antioxidant molecule, and its primary targets in the inner mitochondrial membrane are lipid radicals formed there, one of the main sources of which is cardiolipin, the phospholipid most sensitive to peroxidation. Cardiolipin is an important structural component of various enzyme complexes, and its oxidation leads to the deterioration of the functioning of mitochondria. Being the only negatively charged phospholipid in the mitochondrial membrane, cardiolipin is considered as a primary partner for the interaction with cationic SkQ1, which thus performs its protective antioxidant function. Moreover, SkQ1 and other derivatives of this family were also shown to protect various cells from death induced by exogenous H_2_O_2_, and this protective effect correlated with the decrease in intracellular ROS accumulation [11, 40]. Since in the cited studies SkQ1 selectively accumulated in mitochondria, it was suggested that mtROS were the primary target of SkQ1 antioxidant action. The accumulation of cytoplasmic ROS was observed only as a secondary event after a significant lag-phase as a result of mitochondrial dysfunction and excess ROS production. The SkQ1-dependent decrease in mtROS was proved using the H_2_O_2_-sensitive fluorescent protein HyPer expressed in mitochondria [41] and using a novel mitochondria-targeted dye sensitive to lipid peroxidation [42].

Since in the present study mtAO accumulates in mitochondria of both oligodendrocytes and microglia, as shown in Figures 3(a) and 3(b), it can be assumed that mtAO deactivates mtROS in these cell types. Inasmuch as it has been evidenced that it is peroxynitrite that has a detrimental effect on myelin production by oligodendrocytes [43], it is an important result that mitochondria were abundantly present in the same microglial cells as iNOS molecules and in the myelin-filled oligodendrocytes. This observation points out in favor of the assumption that peroxynitrite can be generated also due to mtROS under pathological conditions; therefore, we cannot exclude the direct effect of mtAO on oligodendrocytes where mtAO scavenges ROS and consequently reduces the possibility of peroxynitrite formation. What else needs attention is that, due to its lipophilic nature, mtAOs are more soluble in the lipid phase and, in particular, SkQ1 concentration can be up to four orders of magnitude higher in lipid structures compared to the aqueous phase in accordance with the membrane/water distribution coefficient [44]. In addition, the plasma membrane is charged and maintains Δ*ψ* of about 60 mV across itself with a negative cell interior, which allows electrophoretic accumulation of a monovalent SkQ1 cation in the cytoplasm by a factor of 10. Therefore, it is reasonable to assume that SkQ1, although accumulating in fatty myelin at concentrations of about 3-4 orders lower than that in mitochondria (in the latter, the magnification of SkQ1 concentration reaches 10^8^ [12]), can also provide antioxidant defense there, as in the case with cardiolipin (see the preceding paragraph). This effect is especially likely in the presence of a reductant or in close vicinity of mitochondria, where mtAO can be constantly recharged into an effectively working, reduced form.

**Figure 4:**
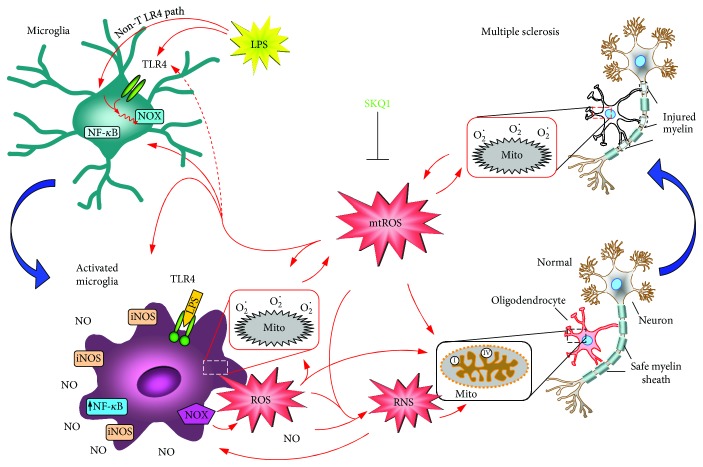
The hypothetic scheme of events induced by LPS in the primary oligodendrocyte culture of cerebellar explants considering the published data. Shown are oligodendrocytes in normal (pink) and pathological (white) states, LPS-initiated microglia (olive) transformed into activated microglia (dark lilac), and the expressed iNOS enzyme producing NO that activates the NOX enzyme (magenta pentagon) making the initial burst of ROS. The appearance of NO and ROS results in generating RNS; mitochondria (framed) with the indicated respiratory complexes I and IV, which mainly produce ROS in them after being toxified by RNS [45]. Blue arrows show transitions from normal to pathological states. Antioxidant SkQ1 (light-green) scavenges ROS and thus decreases the microglia activation, earlier identified as a critical factor contributing to multiple neurodegenerative diseases. For details, see the text.

Within the framework of the in vitro MS model used by us, a hypothetic scheme of the events induced by LPS in the primary oligodendrocyte culture of the cerebellum, containing microglia, is presented in Figure 4.

This chain of events, typical of oxidative stress, includes NF-*κ*B stimulation leading to the induction of proinflammatory cytokines, chemokines, and other mediators [46, 47]. Oxidative stress is implicated in many neurological diseases, including MS [48]. In this context, it is interesting to note that along with neuroprotective action [2, 13–16], SkQ1 and its mtAO analogs demonstrated a pronounced anti-inflammatory effect in the models of local acute inflammation [49] and systemic inflammatory response syndrome (SIRS) [50]. Recently, it was demonstrated that SkQ1 suppressed NF-*κ*B-dependent gene expression in the aortas of old mice [51] and SIRS mice [50]. SkQ1 also inhibited activation of NF-*κ*B, which was induced by tumor necrosis factor in endothelial cells in vitro [51]. These findings indicate that the inhibition of NF-*κ*B by SkQ1 in the LPS-stimulated microglia could contribute to oligodendrocyte protection against demyelination shown by us in the present study.

NADPH oxidase (NOX2) is another participant of the oxidative stress events that produces significant amounts of extracellular ROS, which could play a critical role in the neuroinflammation accompanying MS [52, 53]. Regarding our results, the protective effect of SkQ1 in relation to myelin content in oligodendrocytes (Figure 2) could be explained by the anti-inflammatory action of SkQ1 in microglia, just as SkQ1 prevented NOX2 activation (oxidative burst) recently observed in neutrophils [34].

Also, it is obvious that a decrease in mtROS in activated microglia in the presence of NO produced by iNOS [20] should result in the reduction of RNS generation and thus provide protection to oligodendrocytes in MS [20, 54–56]. Our findings cross-talk with a recent report that RNS production by activated macrophages plays a crucial role in axon demyelination and peripheral neuropathy in leprosy [57]. The demyelination in this mycobacterial disease is analogous to that in MS, and RNS-dependent destruction of mitochondria was suggested to contribute to demyelination in both cases [57]. If this is the case, the mitochondria-targeted antioxidants such as SkQ1, which proved to be effective in the MS model in our experiments, could be considered as a potential medicine against mycobacterial pathogens. The obtained results on the protective effect of the SkQ1 antioxidant are especially relevant in light of the latest data on the accumulation pressure of proinflammatory stimuli on the statistical manifestation of MS [58]. As demonstrated, such incentives include heterogeneous factors leading to inflammatory reactions: smoking, working with organic solvents, and the presence of leukocyte antigen genes in the human genotype. All this means that taking antioxidants that lower the level of ROS, RNS, and inflammation should reduce the risk of MS.

In the context of intractable immunoinflammatory diseases, the results of our study offer an important potentiality for using the antioxidant SkQ1 in an alternative therapy when immunotherapy is not applicable. Recently, a number of immunomodulating, disease-modifying therapies have been introduced, which are aimed at reducing the inflammatory response in MS. Such treatments in varying degrees weaken immune surveillance and may predispose patients to the development of various opportunistic infections that are rarely seen in neurological practice [59]. Among the immunosuppressive drugs, natalizumab, an FDA-approved humanized monoclonal antibody against *α*4-integrin, is considered one of the most effective ways to prevent relapse. Its effect is based on the suppression of both the adhesion of leukocytes (except neutrophils) to the endothelium and their penetration through the blood-brain barrier. However, the high risk of progressive multifocal leukoencephalopathy (PML) limits the long-term treatment with natalizumab. PML can be induced by the John Cunningham virus (JCV), a type of polyomavirus that becomes pathogenic only in cases of immunodeficiency (as in AIDS) or drug-induced immunosuppression [60]. Another immunosuppressive drug, fingolimod, is considered mainly for the postnatalizumab therapy for MS, but its use may increase the incidence of varicella-zoster virus infection [61]. In contrast to immunomodulatory drugs, mitochondria-targeted antioxidants did not cause any signs of immunosuppression in various studies in animal models [12–16]; therefore, their use for MS therapy would presumably be free from the described complications.

## 4. Conclusion

Our study is the first demonstration of the protective effect of the mtAO SkQ1 on oligodendrocytes affected by MS at the level of the culture model of the disease. The pronounced protective action of SkQ1 clearly demonstrates that mtROS participate in the suppression of myelin production in oligodendrocytes. Supporting recent studies [58], we assume that people at risk should take antioxidants to reduce the probability of MS from rising. Also, our results implicate that mtAO could be promising components of a combined therapy for MS, especially in patients with various opportunistic infections accompanying MS, as well as in some disastrous diseases involving neurological disorders.

## Data Availability

The experimental data used to support the findings of this study are included within the article.

## Abbreviations

MS: Multiple sclerosis
mtROS: Reactive oxygen species produced in mitochondria
mtAO: Mitochondria-targeted antioxidants
SkQ1: 10-(6′-Plastoquinonyl) decyltriphenylphosphonium
SkQR1: 10-(6′-Plastoquinonyl) decylrhodamine-19
LPS: Lipopolysaccharide
MBP: Myelin basic protein
NF-*κ*B: Proinflammatory nuclear factor-*κ*B
RNS: Reactive nitrogen species.

## Supplementary Materials

Supplementary Materials Figure S1: myelin content diminished in the cultures after incubation with LPS. The primary oligodendrocyte culture of cerebellar explants of newborn rats upon 14 days of cultivation. Representative images of immunostaining for MBP: (a, c) control and (b, d) treatment with LPS. Number of cells in the field views: (a) 44, (b) 45, (c) 34, and (d) 36. The cells were incubated with rabbit antibodies to MBP and Alexa Fluor 488-conjugated anti-IgG to mark myelin (green). Scale bar, 20 *μ*m. Confocal microscope, Carl Zeiss LSM 510.
