# Supplementary material for: Therapeutic Effect of the Mitochondria-Targeted Antioxidant SkQ1 on the Culture Model of Multiple Sclerosis
Source: Oxid Med Cell Longev. 2019 Jul 1;2019:2082561. doi: 10.1155/2019/2082561 (PMC6636568; doi:10.1155/2019/2082561)
Supplement: Supplementary Materials — Figure S1: myelin content diminished in the cultures after incubation with LPS. The primary oligodendrocyte culture of cerebellar explants of newborn rats upon 14 days of cultivation. Representative images of immunostaining for MBP: (a, c) control and (b, d) treatment with LPS. Number of cells in the field views: (a) 44, (b) 45, (c) 34, and (d) 36. The cells were incubated with rabbit antibodies to MBP and Alexa Fluor 488-conjugated anti-IgG to mark myelin (green). Scale bar, 20 μm. Confocal microscope, Carl Zeiss LSM 510. [file 2082561.f1.pdf]

Supplementary material

TITLE: Therapeutic effect of the mitochondria-targeted antioxidant SkQ1 on the culture model of multiple sclerosis

RUNNING TITLE: Antioxidant protective effect in multiple sclerosis

Elena K. Fetisova, Maria S. Muntyan, Konstantin G. Lyamzaev, Boris V. Chernyak

Belozersky Institute of Physico-Chemical Biology, Lomonosov Moscow State University, Moscow, Russia

***Keywords**: Multiple sclerosis, oligodendrocyte, demyelination, mitochondria, mitochondria-targeted antioxidant, neuroprotection*

Correspondence: Maria S. Muntyan,

A.N. Belozersky Institute, Lomonosov Moscow State University, 119991 Moscow, Russia;

tel. 7(495)939-5360; fax 7(495)939-3181; e-mail: muntyan@genebee.msu.ru

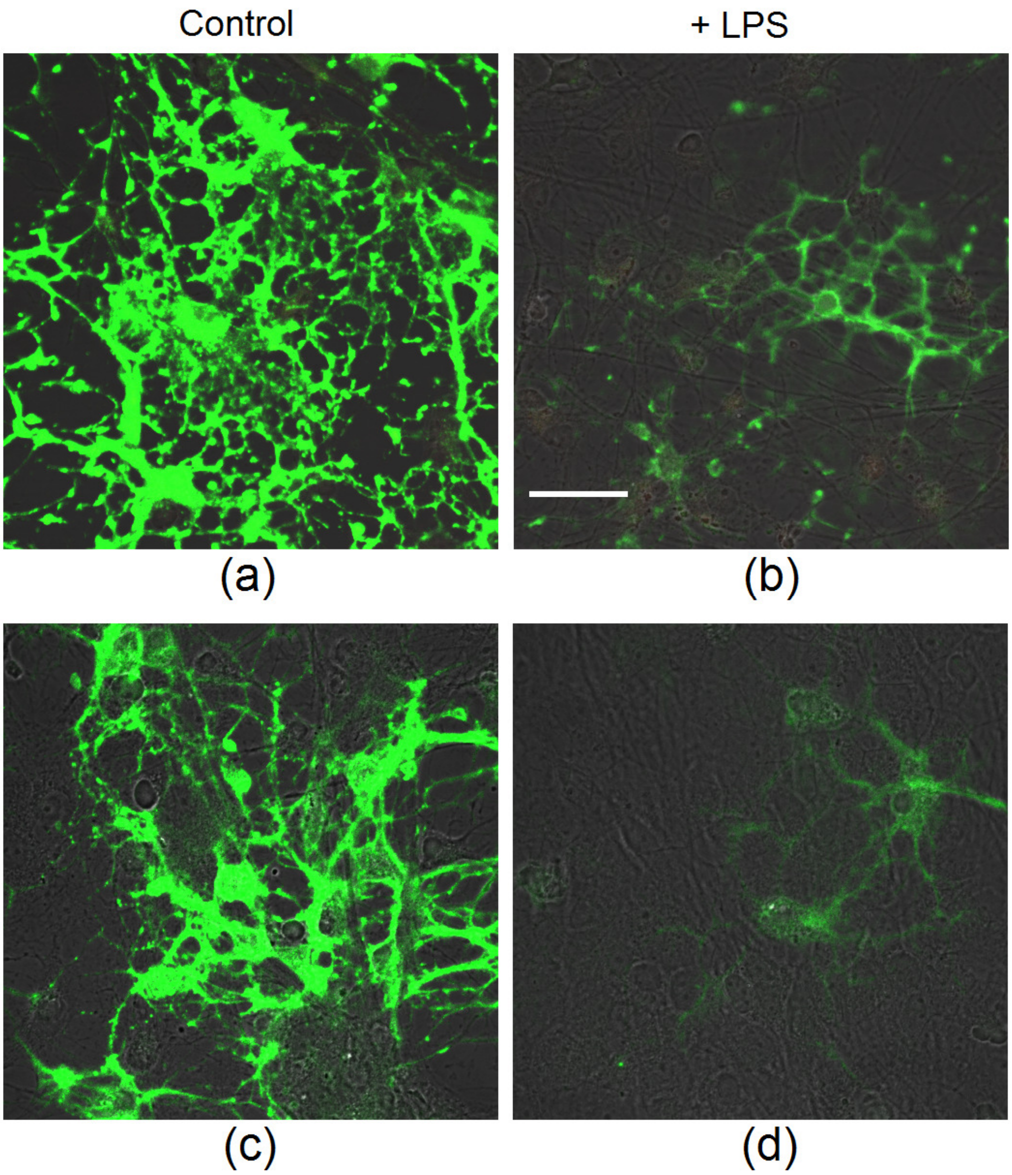

Figure S1. Myelin content was diminished in the cultures after incubation with LPS. The primary oligodendrocyte culture of cerebellar explants of newborn rats upon 14 days of cultivation. Representative images of immunostaining for MBP: (a, c) control and (b, d) treatment with LPS. Number of cells in the field views: (a) 44, (b) 45, (c) 34 and (d) 36. The cells were incubated with rabbit antibodies to MBP and Alexa-Fluor-488-conjugated anti-IgG to mark myelin (green). Scale bar, 20 µm. Confocal microscope, Carl Zeiss LSM 510.
